# Novel mRNA-Engineered Fully Human CAR-T Cells Targeting AXL in Solid Tumors

**DOI:** 10.3390/biomedicines13040844

**Published:** 2025-04-01

**Authors:** Bo Zou, Mengge Wang, Shimeng Bai, Ning Li, Zhongyi Fan, Yuanzheng Peng, Mingshu Han, Chen Zeng, Hongzhou Lu, Lin Qi, Xingding Zhang, Xiaohua Tan, Qibin Liao

**Affiliations:** 1 Shenzhen Key Laboratory for Systems Medicine in Inflammatory Diseases, School of Medicine, Sun Yat-sen University, Shenzhen Campus, Shenzhen 518106, Chinaqilin23@mail.sysu.edu.cn (L.Q.); 2 Biotherapy Clinical Research Center, Shenzhen Third People’s Hospital, The Second Affiliated Hospital to Southern University of Science and Technology, Shenzhen 518112, China; sophia15354815841@163.com (S.B.); lining.yatu@hotmail.com (N.L.); fzyi11@163.com (Z.F.); hanmingshu565@163.com (M.H.); xiaohua_t@126.com (X.T.); 3 Institute of Advanced Biotechnology and School of Medicine, Southern University of Science and Technology, Shenzhen 518055, China; 18614982193@163.com (M.W.); zengc@sustech.edu.cn (C.Z.); 4 Kidney Transplant Department (Liver Transplant Department), Transplant Center, National Clinical Research Center for Infectious Disease, Shenzhen Third People’s Hospital, The Second Affiliated Hospital to Southern University of Science and Technology, Shenzhen 518112, China; pyz668@sina.com; 5 National Clinical Research Centre for Infectious Diseases, Shenzhen Third People’s Hospital, The Second Affiliated Hospital to Southern University of Science and Technology, Shenzhen 518112, China; luhongzhou@fudan.edu.cn

**Keywords:** AXL, mRNA, fully human CAR-T, solid tumors, immunotherapy

## Abstract

**Background/Objectives:** The AXL receptor tyrosine kinase is a promising therapeutic target in solid tumors, yet conventional viral vector-engineered CAR-T cells face critical limitations, including risks of insertional mutagenesis and immunogenicity from murine-derived single-chain variable fragments (scFvs). This study aimed to develop and evaluate mRNA-engineered fully human AXL CAR-T (^mfh^AXL CAR-T) cells as a safer, scalable alternative for solid tumor immunotherapy. **Methods:** ^mfh^AXL CAR-T cells were generated via electroporation-mediated delivery of in vitro transcribed mRNA encoding a fully human AXL-specific CAR. CAR expression kinetics and T-cell viability were quantified by flow cytometry. Antitumor activity was assessed through in vitro co-cultures with AXL-positive lung and pancreatic cancer cells, measuring cytotoxicity, cytokine secretion, and specificity. In vivo efficacy was evaluated in a lung cancer xenograft mouse model, with tumor volume and body weight monitored over 14 days. **Results:** Flow cytometry confirmed transient but high CAR expression (>90% at 24 h) with preserved T-cell viability (>90%). In vitro, ^mfh^AXL CAR-T cells exhibited dose-dependent cytotoxicity and antigen-specific cytokine secretion. In vivo, four administrations of ^mfh^AXL CAR-T cells suppressed tumor growth without body weight loss. **Conclusions:** The mRNA-electroporated ^mfh^AXL CAR-T platform enables cost-effective, large-scale production, offering a safer alternative to viral vector-based approaches by eliminating risks of insertional mutagenesis and immunogenicity.

## 1. Introduction

Cancer poses a significant health threat, ranking second in global mortality after ischemic heart disease [1]. In China, solid tumors dominate cancer cases, with lung cancer topping incidence and mortality lists for both genders [2]. Traditional treatment includes surgery, radiation, and chemotherapy, but they are often prolonged and accompanied by severe side effects. Recent medical advancements have swiftly established immunotherapy as a fourth pivotal treatment modality [3]. Chimeric antigen receptor-T (CAR-T) immunotherapy has revolutionized hematological malignancy treatment but faces challenges in solid tumors due to target antigen scarcity and the immunosuppressive microenvironment [4]. AXL, a receptor tyrosine kinase (RTK), modulates cellular functions upon GAS6 ligand binding [5,6,7,8] and is often upregulated in aggressive, metastatic tumors, which correlates with poor prognosis [9]. Solid tumors feature a complex microenvironment with blood vessels, cancer cells, stromal cells, and immunosuppressive cells, like myeloid-derived suppressor cells (MDSCs) and tumor-associated macrophages (TAMs) [10,11,12,13]. AXL-targeting drugs, including AXL tyrosine kinase inhibitors and AXL/GAS6 blocking antibodies, show promising antitumor activity through reshaping the complex tumor microenvironment (TME) [14,15,16,17]. Moreover, AXL has been demonstrated to exhibit high expression levels across various cancers but is absent in normal tissues, rendering it a potential target for cell-based therapy. Preclinical studies have used viral vector-engineered AXL CAR-T cells, yielding promising outcomes in the treatment of breast, lung, and pancreatic cancers [18,19,20,21].

Viral vector-engineered CAR-T cells have emerged as a promising therapeutic modality in various malignancies, particularly B-cell malignancies. However, the use of viral vectors in the engineering of these cells raises significant concerns regarding insertional mutagenesis and immunogenicity. Insertional mutagenesis occurs when the viral vector integrates into the host genome at random sites, potentially disrupting essential genes or activating oncogenes, which can lead to malignant transformations. Notably, there is increasing incidence of secondary T-cell malignancies among suspected adverse reactions associated with viral vector-engineered CAR-T-cell therapy [22,23]. Immunogenicity is another critical issue associated with viral vector-engineered CAR-T cells. The use of murine-derived single-chain variable fragments (scFvs) in the construction of CARs can trigger immune responses in patients, leading to the rejection of the CAR-T cells and reducing their therapeutic efficacy. This has been observed in clinical trials where patients developed human anti-mouse antibodies (HAMAs) against the murine components of the CAR, compromising the persistence and effectiveness of the therapy [24,25]. To mitigate these risks, researchers have been exploring the development of fully human or humanized CAR constructs, which have shown reduced immunogenicity and improved antitumor effects in preclinical and clinical studies [26,27]. Additionally, alternative non-viral methods for CAR-T-cell engineering are being investigated to address the safety concerns associated with viral vectors. Techniques such as the use of mRNA, transposon systems (e.g., Sleeping Beauty, piggyBac) and CRISPR/Cas9 genome editing offer potential solutions by delivering CAR constructs, thereby reducing the risk of insertional mutagenesis [28]. These approaches also allow for the generation of CAR-T cells with reduced immunogenicity, as they can be engineered to express humanized or fully human CARs without the need for viral components.

Here, we have developed novel mRNA-engineered fully human AXL CAR-T (^mfh^AXL CAR-T) cells to overcome challenges posed by insertional mutagenesis and immunogenicity associated with current viral vector-engineered AXL CAR-T cells. These ^mfh^AXL CAR-T cells were efficiently generated by electroporating mRNA encoding fully human AXL CAR, which was derived from the scFvs of a fully human AXL-specific antibody. We observed that the preparation of ^mfh^AXL CAR-T cells is both simple and rapid, featuring transient yet high-level CAR expression coupled with high viability, facilitating cost-effective large-scale production and avoiding risks of viral vector-driven insertional mutagenesis. The in vitro results demonstrate that the ^mfh^AXL CAR-T cells exhibited robust antitumor activity against tumor cells. Upon interaction with target cells, these cells specifically secreted cytokines IL-2 and IFN-γ while exhibiting minimal cytotoxicity toward non-target cells, underscoring their favorable safety profile. Furthermore, in a lung cancer-bearing mouse model, multiple infusions of ^mfh^AXL CAR-T cells were observed to elicit potent antitumor effects, significantly inhibiting tumor growth without any signs of toxicity as measured by body weight. Collectively, the transient and high-level expression of ^mfh^AXL CAR in T cells enables cost-effective, large-scale production, offering a safer therapeutic alternative for solid tumors that avoids the risks of insertional mutagenesis and immunogenicity associated with current viral vector-engineered AXL CAR-T-cell therapy.

## 2. Materials and Methods

### 2.1. Cell Lines and Cell Culture

Unless otherwise noticed, all cell lines and reagents were obtained from the American Type Culture Collection (ATCC, Manassas, VA, USA). The human cancer cell lines Panc-1 (pancreatic cancer), A549 (non-small cell lung cancer), A549-LUC, and HepG2 (hepatocellular carcinoma) were cultured at 37 °C in a humidified atmosphere of 95% air and 5% CO_2_. These cells were grown in DMEM or RPMI1640 medium (Corning, New York, NY, USA) supplemented with 10% fetal bovine serum (FBS; Corning, USA) and 1% penicillin–streptomycin–neomycin solution (PSN; Yeasen, Shanghai, China).

### 2.2. Ex Vivo Expansion of T Cells

Human peripheral blood mononuclear cells (PBMCs) were isolated from the blood of healthy donors by density gradient centrifugation. The PBMCs were then frozen in liquid nitrogen with a mixture of 90% FBS and 10% DMSO until further use. After thawing, the PBMCs were resuscitated in serum-free medium X-VIVO15 (Lonza, Morristown, NJ, USA) containing 50 IU/mL interleukin-2 (IL-2), 5 ng/mL recombinant human interleukin-7 (IL-7) and 10 ng/mL interleukin-15 (IL-15) (Novoprotein, Shanghai, China) and allowed to stand for 2–4 h in a T25 flask to remove adherent cells at 37 °C and 5% CO_2_. After 2–4 h of resting, the suspended cells in the bottle were aspirated, centrifuged, and subsequently resuspended to a volume of 5 mL for counting. Magnetic beads coated with human CD3/CD28 agonist antibody were then added at a cell-to-bead ratio of 1:1 to specifically activate and expand T cells within PBMCs. The culture was maintained for 7–10 days to yield a sufficient number of T cells for the generation of ^mfh^AXL CAR-T cells.

### 2.3. CAR Construct and mRNA Preparation

The fully human AXL CAR consists of CD8 signal peptide, FLAG tag, fully human AXL-specific scFvs derived from the European Patent (EP 4357365 A1) [29], CD8 hinge region, CD8 transmembrane region, 4–1BB cytoplasmic domain, and CD3ζ. Briefly, the AXL-specific scFvs were isolated from a fully human phage display library using iterative rounds of in vitro panning against recombinant human AXL protein. This process yielded high-affinity, fully human scFvs, which were further codon-optimized and incorporated into the CAR construct. The nucleotide and amino acid sequences of the fully human AXL CAR are provided in Supplementary Table S1. The mRNA utilized in this study was derived from our previously optimized non-amplifying mRNA platform [30]. The mRNA was synthesized by T7 RNA polymerase using a linearized plasmid encoding a codon-optimized AXL-specific CAR (synthesized by Sangon Biotech, Shanghai, China). This plasmid included a 5′ untranslated region (5′ UTR), a 3′ UTR, and a 250 poly-A sequence, thereby obviating the need for poly(A) tailing with poly(A) polymerase during in vitro transcription (IVT). N1-methyl-pseudouridine (m1Ψ, Nanjing Synthgene Medical Technology Co., Ltd., Nanjing, China) was utilized in place of UTP to synthesize mRNA incorporating a modified nucleoside aimed at enhancing stability. Subsequently, the in vitro transcribed mRNAs were capped using the Vaccinia Capping System and an mRNA Cap 2′-O-methyltransferase (Novoprotein, China). The mRNA was precipitated overnight with 2.5 M LiCl at −20 °C, followed by centrifugation at maximum speed. The resulting mRNA pellets were subsequently washed with 70% ethanol and resuspended in RNase-free water. The nucleoside-modified mRNAs were characterized using agarose gel electrophoresis and capillary electrophoresis and stored at −20 °C for further use.

### 2.4. Generation of ^mfh^AXL CAR-T Cells Through Electroporation

**Figure 1:**
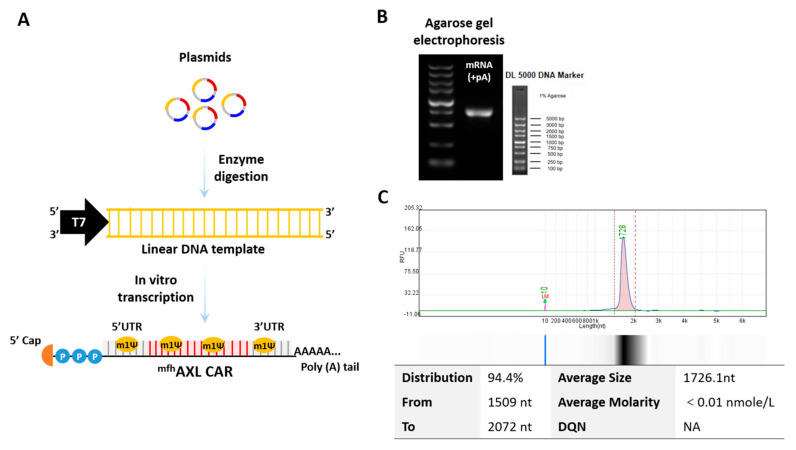
**Production of mRNA encoding ^mfh^AXL CAR.** (**A**) The circular supercoiled plasmid undergoes enzymatic digestion to yield a linear DNA template incorporating the T7 promoter sequence. This template serves as the basis for the synthesis of mRNAs with N1-methylpseudouridine (m1Ψ) modifications, followed by the addition of a poly(A) tail through in vitro transcription (IVT) and the addition of a 5′ cap structure using the Vaccinia Capping System. (**B**) A representative agarose gel electrophoresis image illustrating AXL-specific CAR mRNA. (**C**) A representative capillary electrophoresis image obtained using Qsep, depicting the distribution of ^mfh^AXL CAR mRNA.

As shown in Figure 1A, 1 × 10^6^ activated T cells were transfected with the indicated dose of ^mfh^AXL CAR mRNA to generate ^mfh^AXL CAR-T cells, which was conducted via electroporation using the Celetrix Electroporation system (Celetrix Corporation, Manassas, VA, USA) under optimized parameters: volume, 20 μL; voltage, 500 V; pulse length, 20 ms; a single pulse. Subsequently, these T cells were incubated in a humidified environment at 37 °C with 5% CO_2_ for at least 6 h prior to downstream experiments.

### 2.5. Cytotoxicity Analysis of CAR-T Cells

Unless otherwise stated, all CAR-T-cell analyses were performed following a 6-h resting period post-electroporation. For the cytotoxicity analysis of CAR-T cells, the Cell Counting Kit-8 reagent (CCK-8; MCE, San Rafael, CA, USA) was employed to evaluate the cytolytic activity against all cell lines. Target cells (1.5 × 10^4^ cells each) and CAR-T cells were seeded in a flat-bottom 96-well plate (100 μL volume) at indicated effector-to-target (E/T) ratios and incubated for 24 h at 37 °C with 5% CO_2_. The effector cells were co-incubated with the target cells for a duration of 24 h. Following incubation, the supernatant was discarded, and the cells were washed twice with 1 × PBS. Subsequently, 100 μL of medium was added to each well, followed by the addition of 10 μL of CCK-8 reagent. The optical density at a wavelength of 450 nm was then measured using the Varioskan LUX Multimode Microplate Reader (ThermoFisher Scientific, Waltham, MA, USA). Control groups were established to include a target group (only target cells) and a medium group (no cells). The proportion of cytotoxicity was quantified by evaluating the fraction of live cells. The percentage of specific lysis (as cytotoxicity (%)) was determined using the following formula: Cytotoxicity (%) = (1 − (Experimental group (OD) − Medium group (OD))/(Target group (OD) − Medium group (OD))) × 100.

### 2.6. Cytokine Secretion Assay by ELISA

Supernatants from co-cultures with an E/T ratio of 4:1 were collected over a 24-h period, subjected to high-speed centrifugation, and subsequently transferred to new centrifuge tubes for further analysis. The Human IL-2 and IFN-γ Precoated ELISA Kit (Dakewe Biotech Co., Ltd., Shenzhen, China) was then utilized according to the manufacturer’s instructions.

### 2.7. Flow Cytometric Analysis

The expression of the AXL antigen, ^mfh^AXL CAR, and cell viability were analyzed using flow cytometry on the BD Symphony A3 (BD Biosciences, Franklin Lakes, NJ, USA). The following reagents were utilized: PE-AXL (clone: DS7HAXL, Invitrogen, Waltham, MA, USA), PE-FLAG tag (clone: L5, Biolegend, San Diego, CA, USA), and Live/Dead (Zombie NTR, Biolegend, San Diego, CA, USA). One million cells were harvested from cell culture flasks and subsequently washed twice with cold FACS Buffer (PBS containing 0.02% FBS). These cells were then pelleted by centrifugation at 400× *g* for 5 min. Following this, these cells were stained with various antibodies, depending on the assay’s purpose, for a duration of 20 min at room temperature (RT). The cells were then washed once with FACS buffer, resuspended in 300 μL of FACS buffer, and subjected to flow cytometry acquisition using the indicated gating strategies (Supplementary Figure S1). The acquired data were subsequently analyzed using Flowjo v10.0 software.

### 2.8. In Vivo Animal Experiment

All animal procedures were approved by the Institutional Animal Care and Use Commission of The Third People’s Hospital of Shenzhen and were conducted in accordance with the approved protocol. A549-LUC lung cancer cells (2 × 10^6^) were subcutaneously inoculated into 6–8-week-old female NSG mice, which were purchased from GemPharmatech Co., Ltd., (Nanjing, China). The inoculated NSG mice were randomly assigned to the following three experimental groups (4–5 mice per group): vehicle, control T cells, and ^mfh^AXL CAR-T cells. Seven days following the subcutaneous infusion of tumor cells, 5 × 10^6^ CAR-T cells or control T cells were administered intravenously to the tumor-bearing mice. The same dose of ^mfh^AXL CAR-T cells or control T cells was subsequently infused every three days, with the treatment regimen continuing for a total of four administrations. Prior to each treatment, the mice were weighed, and the tumor size was documented through photography and bioluminescence imaging. Additionally, the tumor volume was measured using calipers and calculated with the following formula: tumor volume = (length × width^2^)/2. At the end of the experiment, the mice were euthanized, and the tumors were excised and subsequently weighed.

### 2.9. Statistical Analyses

Statistical analyses were performed using GraphPad Prism version 8 software. Student’s *t*-test was employed to assess the statistical significance of differences between two groups, while one-way ANOVA was applied for comparisons involving three or more groups. All values with *p* < 0.05 were considered significantly different, where * *p* < 0.05, ** *p* < 0.01, *** *p* < 0.001, and **** *p* < 0.0001.

## 3. Results

### 3.1. Features of mRNA Encoding ^mfh^AXL CAR

An mRNA encoding ^mfh^AXL CAR was synthesized via in vitro transcription (IVT) using linear DNA templates obtained through enzymatic digestion. The ^mfh^AXL CAR is composed of the signal peptide of CD8, fully human AXL-specific scFvs, the hinge region of CD8, the transmembrane region of CD8, the co-stimulatory domain of d 4–1BB, and the CD3ξ signaling domain (Supplementary Table S1). In addition to a poly(A) tail, the mRNA features a cap 1 structure at the 5′ end and is fully modified with N1-methyl-pseudouridine (m1Ψ) (Figure 1A). The expression of the mRNA was assessed using agarose gel (Figure 1B) and capillary electrophoresis (Figure 1C), and the results confirm the full-length product, integrity, and purity of the mRNA.

### 3.2. High Performance of ^mfh^AXL CAR mRNA Delivery Using Electroporation

**Figure 2:**
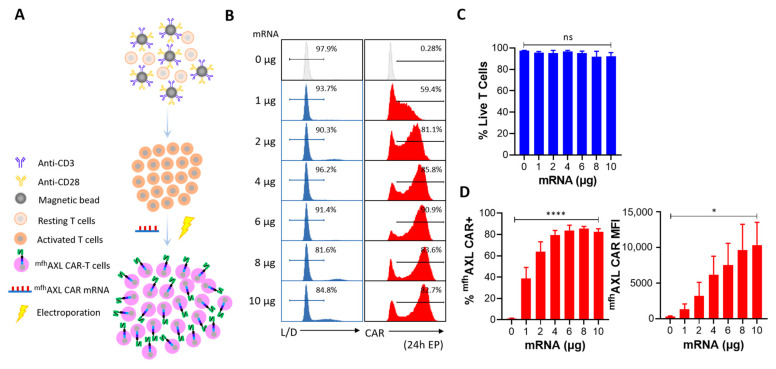
**Screening for optimal mRNA electroporation dose.** (**A**) Human PBMCs were isolated and subsequently activated using CD3/CD28 agonist antibody-coated magnetic beads. Seven to ten days later, electro-transfer of ^mfh^AXL CAR-encoded mRNA was performed at indicated concentrations of mRNA per 10^6^ cells. (**B**) Representative flow cytometry histograms illustrate cell viability and CAR expression on day 1 post-electroporation of T cells (CAR-T, red) in comparison to untransfected T cells (control T, grey). Cell viability was assessed using a Live/Dead dye reagent, and CAR expression was analyzed through staining with PE-FLAG tag antibody. (**C**) Various doses of mRNA electroporation were assessed for cell viability using Live/Dead dye one day post-transfection (mean ± SEM, n = 3 independent experiments/donors). (**D**) The expression rate of ^mfh^AXL CAR (**left**) and the mean fluorescence intensity (MFI) level (**right**) were measured one day after EP across different mRNA doses (mean ± SEM, n = 3 independent experiments/donors). Statistical significance was determined using one-way ANOVA: ns, not significant; * *p* < 0.05; **** *p* < 0.0001.

As shown in Figure 1A, the Celetrix Electroporation system was employed to facilitate electroporation, enabling the manual adjustment of pulse voltage in two different modes, including cell line mode and PBMC mode, to simplify experimental procedures. The activated T cells were resuspended in electroporation buffers A and B, provided as part of the kit, and subjected to electroporation at 500 V, 20 ms, with a single pulse. Subsequently, the indicated dose of ^mfh^AXL CAR mRNA was introduced into activated T cells via electroporation, after which these cells were cultured for further usage. Additionally, these cells were harvested and incubated with PE-FLAG antibody and Live/Dead dye reagents to assess the surface ^mfh^AXL CAR expression and cell viability, and the gating strategies were observed (Supplementary Figure S1). The results show that the dose of mRNA electroporation had a certain effect on the T-cell viability. Specifically, as the mRNA dosage increased, the viability of the ^mfh^AXL CAR-T cells progressively decreased. Notably, when the dose of mRNA electroporation exceeded 6 μg, cell viability decreased to below 90% (Figure 2B,C). Analysis of the ^mfh^AXL CAR-T cells revealed a relatively high expression of ^mfh^AXL CAR at a 2 μg dose of electroporation, indicative of a rapid and efficient mRNA transfer mechanism. The peak ^mfh^AXL CAR expression rate was observed at a 6 μg electroporation dose, followed by a gradual decline with further dose increases (Figure 2B,D). Conversely, the mean fluorescence intensity (MFI) of ^mfh^AXL CAR, serving as a measure of intensity of CAR expression, exhibited an upward trend with increasing mRNA electroporation doses (Figure 2D). Based on the aforementioned findings, an optimal dose of 6 μg of mRNA per 10^6^ T cells was determined to achieve high CAR expression while preserving cell viability using electroporation.

### 3.3. The Expression and Functional Dynamics of ^mfh^AXL CAR-T Cells

**Figure 3:**
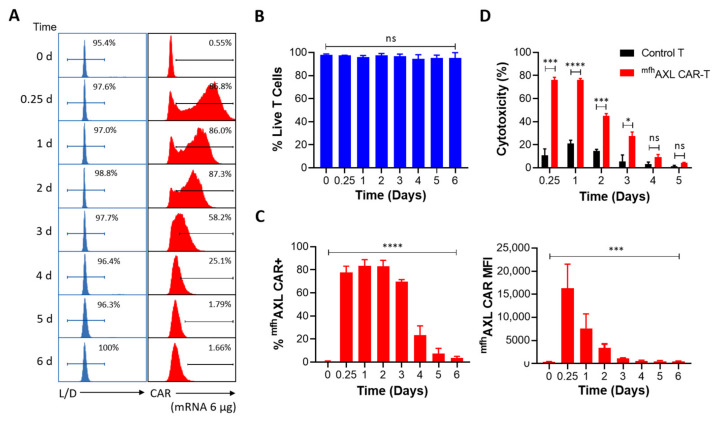
**Expression and functional dynamics of ^mfh^AXL CAR-T.** Human T cells were transfected with ^mfh^AXL CAR-encoded mRNA to generate CAR-T cells via EP, while untransfected T cells served as a negative control for comparative analysis. (**A**) Representative flow cytometry histograms illustrate T-cell viability and CAR expression from day 0 to day 6 post-EP, compared to untransfected T cells. T-cell viability was assessed using a Live/Dead reagent, while CAR expression was analyzed using PE-FLAG tag staining. (**B**) Statistical histogram of T-cell viability from day 0 to day 6 post-EP, presented as mean ± SEM, with data collected from three donors (n = 3). (**C**) Statistical histograms depicting the percentage of ^mfh^AXL CAR-positive cells (left panel) and the median fluorescence intensity (MFI) of ^mfh^AXL CAR (right panel) from day 0 to day 6 post-EP (mean ± SEM, n = 3 donors). Statistical significance was determined using one-way ANOVA: ns, not significant; *** *p* < 0.001; **** *p* < 0.0001. (**D**) The functionality of ^mfh^AXL CAR-T cells was assessed by their cytotoxicity against AXL-expressing A549 cells in a co-culture (E/T ratio = 4:1 for 24 h) from 0.25 to 5 days post-EP, compared to untransfected (UTF, black) T cells (mean ± SEM of n = 3 independent experiments/donors). Statistical significance was determined using Student’s *t*-test. ns, not significant; * *p* < 0.05; *** *p* < 0.001; **** *p* < 0.0001.

We have determined that 6 μg of mRNA is the optimal dose for electroporation to generate ^mfh^AXL CAR-T cells. Over a period of six consecutive days, we monitored the viability and CAR expression of ^mfh^AXL CAR-T cells post-electroporation. The results indicate no significant alteration in the viability of ^mfh^AXL CAR-T cells produced using a 6 μg electroporation dose, thereby fully satisfying the requisites for subsequent experimental procedures (Figure 3A,B). Additionally, high-intensity ^mfh^AXL CAR expression was observed in T cells at 6 h (0.25 days) post-electroporation. ^mfh^AXL CAR expression peaked on the first day post-electroporation and maintained elevated levels for two days before gradually declining to less than 10% by the fifth day post-electroporation (Figure 3A,C). However, the MFI of ^mfh^AXL CAR, indicative of the expression intensity, was already reduced by day 1 post-electroporation. From that point, the MFI decreased rapidly, falling below 1000 by day 3 post-electroporation (Figure 3C). In addition to assessing the viability and sustained CAR expression of ^mfh^AXL CAR-T cells, we evaluated their cytotoxic activity against AXL-positive A549 cells (Supplementary Figure S2) over a consecutive five-day period post-electroporation (Figure 3D). Consistent with the CAR expression and MFI levels, the kill rate of ^mfh^AXL CAR-T cells was approximately 80% at 0.25 days and 1 day post-electroporation. However, the antitumor efficacy of ^mfh^AXL CAR-T cells significantly declined to about 10% by day 4, which correlated with the observed trends in CAR expression and MFI changes.

### 3.4. ^mfh^AXL CAR-T Cells Exhibit Cytotoxic Activity Against Various Cancer Cell Lines and Demonstrate Specific Cytokines Secretion

**Figure 4:**
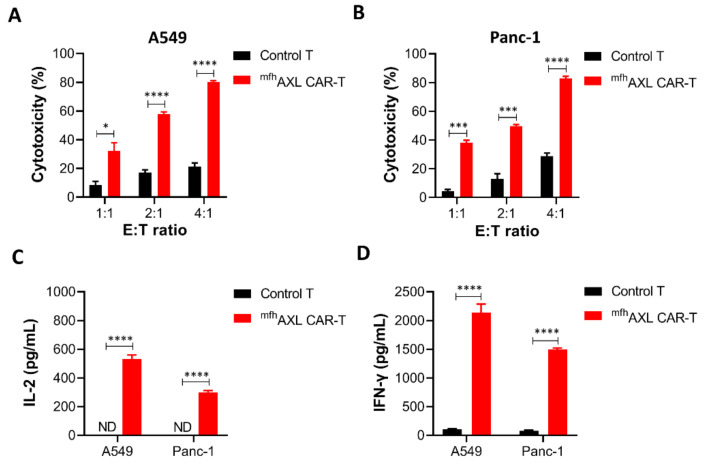
**Antitumor effects and cytokine secretion of ^mfh^AXL CAR-T cells in solid tumor cells.** The ^mfh^AXL CAR-T cells demonstrated cytotoxic activity against lung and pancreatic cancer cells, exhibiting increased secretion of IL-2 and IFN-γ compared to untransfected T cells. Specifically, cytotoxicity assays were performed using ^mfh^AXL CAR-T cells against lung cancer A549 cells (**A**) and pancreatic cancer Panc-1 cells (**B**). The secretion levels of IL-2 (**C**) and IFN-γ (**D**) were assessed in ^mfh^AXL CAR-T cells targeting A549 and Panc-1 cells, with an E/T ratio of 4:1. The bar chart represents the mean values obtained from three independent experimental measurements. * *p* < 0.05; *** *p* < 0.001; **** *p* < 0.0001, comparing ^mfh^AXL CAR-T to control T cells using Student’s *t*-test.

To evaluate the antitumor efficacy of ^mfh^AXL CAR-T cells in the treatment of solid tumors, we conducted a cytotoxicity assay using ^mfh^AXL CAR-T and untransfected T cells (control T) against AXL-positive lung cancer cells (A549) and pancreatic cancer cells (Panc-1) at different E/T ratios (Supplementary Figure S2). These cell mixtures were incubated for 24 h, followed by assessment using the CCK8 reagent. The results demonstrate that ^mfh^AXL CAR-T cells exhibited a dose-dependent cytotoxic effect against A549 cells, significantly surpassing the efficacy of control T cells (Figure 4A). Furthermore, consistent results were observed for ^mfh^AXL CAR-T cells when co-cultured with Panc-1 cells (Figure 4B). Given the potential for CAR-T cells to induce off-target cytotoxicity in antigen-negative normal tissue cells during the treatment of solid tumors, we evaluated the off-target effects by testing the ^mfh^AXL CAR-T cells against AXL-negative HepG2 cells (Supplementary Figure S3A). Compared to the control T cells, the ^mfh^AXL CAR-T cells did not exhibit significant cytotoxicity after one day of co-incubation with HepG2 at various E/T ratios (Supplementary Figure S3B). To examine the cytokine secretion by ^mfh^AXL CAR-T cells during the antitumor response, supernatants were collected at a 4:1 E/T ratio following the killing assay. Subsequently, the cytokine levels were quantified using ELISA after co-culturing CAR-T cells with different target cell types. The results show that the ^mfh^AXL CAR-T cells specifically secreted elevated levels of interleukin-2 (IL-2) and interferon-γ (IFN-γ) when co-cultured with A549 and Panc-1 cancer cells, as compared to control T cells, which indicate specific T-cell activation in the presence of target cells (Figure 4C,D).

### 3.5. ^mfh^AXL CAR-T Cells Exhibit Potent In Vivo Anti-Tumor Efficacy

**Figure 5:**
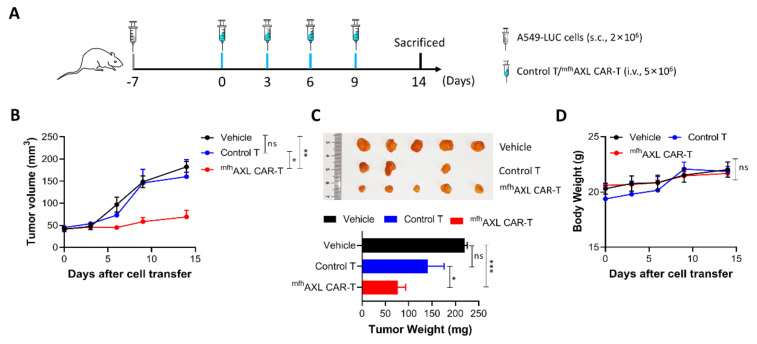
**Antitumor efficacy of ^mfh^AXL CAR-T cells in subcutaneous lung cancer xenograft models.** NSG mice were inoculated and subsequently randomized into the following experimental groups: vehicle group (black), control T-cell group (blue), and ^mfh^AXL CAR-T-cell group (red). (**A**) Experimental design for tumor-bearing mice. NSG mice were subcutaneously injected with 2 × 10^6^ A549-LUC cells. Upon reaching a tumor volume of approximately 50 mm^3^, the mice received tail vein injections of 5 × 10^6 mfh^AXL CAR-T cells or control T cells every three days. (**B**) The tumor sizes were monitored by caliber prior to each intravenous injection of T cells. The tumor volume was quantified in each mouse. (**C**) At the end of the experiment, the mice were euthanized, and the tumors were excised and weighed. (**D**) The mice were weighed before each injection of T cells. Statistical significance was determined using one-way ANOVA: ns, not significant; * *p* < 0.05; ** *p* < 0.01; *** *p* < 0.001.

Following the validation of the in vitro experiments, we proceeded to assess the in vivo antitumor activity of the ^mfh^AXL CAR-T cells. Concurrently, to determine whether ^mfh^AXL CAR-T cells could inhibit the growth of AXL-positive lung cancer, we established a subcutaneous tumor model by injecting mice with luciferase-overexpressing A549 cells (A549-LUC). One-week post-inoculation, we commenced the infusion of ^mfh^AXL CAR-T cells at three-day intervals (Figure 5A). The inoculated NSG mice were then randomly allocated into three groups: the vehicle group, the control T-cell group, and the ^mfh^AXL CAR-T-cell group. Seven days after tumor inoculation via subcutaneous injection, tumor cells were detected using bioluminescence imaging. On the same day, 5×10^6^ control T cells or ^mfh^AXL CAR-T cells were administered intravenously into tumor-bearing mice, with subsequent doses of control T cells or ^mfh^AXL CAR-T cells given every three days. The tumor volume monitoring data demonstrate that the ^mfh^AXL CAR-T cells effectively inhibited tumor growth, in contrast to the control T cells or vehicle treatment, which did not impede tumor progression (Figure 5B). These findings are corroborated by the in vivo bioluminescence imaging of transplanted tumors (Supplementary Figure S4). Furthermore, the tumor weight measured in the ^mfh^AXL CAR-T-cell group was significantly lower compared to that in the control T-cell and vehicle groups (Figure 5C). In addition, mouse body weights were recorded at each imaging session, revealing no significant difference between the groups (Figure 5D), and indicating a favorable safety profile of ^mfh^AXL CAR-T cells.

## 4. Discussion

CAR-T-cell therapy has demonstrated remarkable efficacy in the treatment of hematological malignancies. Nevertheless, its application in solid tumors has yet to yield satisfactory clinical outcomes, underscoring the need for further research advancements [31]. Recently, Maalej et al. provided a comprehensive summary of clinical outcomes associated with numerous CAR-T-cell therapies for solid tumors. These therapies were categorized according to the targeted tumor antigens, highlighting the extensive potential of CAR-T cells in treating certain solid tumors. Nonetheless, several limitations were also identified, notably on-target off-tumor toxicity, and cytokine release syndrome (CRS), which are often attributed to the absence of specific tumor antigens [32]. Consequently, the identification of novel and appropriate tumor target antigens is crucial for advancing the CAR-T-cell therapy of solid tumors. AXL is aberrantly upregulated in the majority of aggressive and metastatic human tumors, particularly across various types of solid tumors, whereas its expression remains low or absent in normal tissues [9]. Therefore, AXL has emerged as a promising tumor target antigen for the development of CAR-T-cell therapy, which has demonstrated encouraging preclinical therapeutic results [18,19,20,21]. Currently, the generation of CAR-T cells primarily relies on the in vitro modification of T cells through the genomic integration of retrovirus (RV) or lentivirus (LV). However, this approach raises significant safety concerns due to the use of viral vectors and poses numerous regulatory hurdles for clinical application [28]. Furthermore, the production of a sufficient number of adoptive CAR-T cells via viral vector transduction is a time-consuming and costly process. Numerous studies have also indicated that viral vector-engineered CAR-T-cell therapy may lead to an increased incidence of suspected adverse reactions, such as secondary T-cell malignancies [22,23]. With the ongoing advancements in immuno-oncology research, several non-viral vector technologies have been developed to facilitate the genetic engineering of T cells. Among these technologies, mRNA-engineered CAR-T-cell manufacturing stands out as a safer alternative to viral vector-engineered CAR-T cells, which facilitates the non-integrated modification of T cells, thereby producing CAR-T cells that eliminate the risks of insertional mutagenesis and exhibit reduced immunogenicity due to the incorporation of modified nucleoside in the non-integrated mRNA [33,34]. Moreover, immunogenicity represents a significant concern in the application of viral vector-engineered CAR-T cells. The incorporation of murine-derived scFvs in the design of CARs can elicit immune responses in patients, potentially resulting in the rejection of CAR-T cells and a consequent decrease in their therapeutic efficacy. This issue may be mitigated by replacing fully human or humanized scFvs.

Therefore, we aimed to develop novel mRNA-engineered fully human AXL CAR-T (^mfh^AXL CAR-T) cells for treating solid tumors, circumventing the risks of insertional mutagenesis and immunogenicity. Our study revealed that the ^mfh^AXL CAR-T cells demonstrated robust AXL-specific CAR expression as early as 0.25 days post-electroporation and maintained high antitumor cytotoxic activity while minimally affecting T-cell viability. These results are consistent with those reported in previous studies. The observed high viability of mRNA-engineered CAR-T cells may be attributed to the initial high viability of T cells (≥90%) or the different electroporation system utilized in our research [35,36]. The cytotoxic lifespan of mRNA-engineered CAR-T cells exhibited a gradual decline within 4 days of mRNA electroporation into T cells, suggesting a potential correlation between the CAR expression levels and the duration of cytotoxicity against the target cancer cells [35,37]. This observation aligns with our findings that the ^mfh^AXL CAR-T cells exhibited transiently elevated levels of CAR expression, which progressively decreased over time and became undetectable by day 6 post-electroporation. Additionally, the in vitro cytotoxicity was confined to a maximum duration of 4 days, primarily due to the inherent instability and susceptibility to degradation of mRNA. These findings underscore that the ^mfh^AXL CAR can be efficiently introduced into activated T cells via mRNA electroporation, resulting in transient CAR expression on the T-cell surface. Consequently, cancer immunotherapy employing mRNA-engineered CAR-T cells often requires a large number of T cells and repeated infusions to satisfy therapeutic demands [38,39]. The expression of CAR following mRNA electroporation is influenced by various factors, including mRNA synthesis, mRNA design, and electroporation conditions [40]. The strategies employed for mRNA delivery are pivotal in determining the duration of CAR expression. Notably, the delivery of lipid nanoparticles (LNPs) could significantly prolong the persistence of CAR-mRNA and CAR expression compared to electroporation (6 days vs. 4 days). This extension is attributed to the different delivery mechanism of LNPs, which also resulted in the reduced cytotoxicity and slower proliferation of CAR-T cells [35]. These findings underscore the substantial potential of mRNA-LNP delivery for ex vivo ^mfh^AXL CAR-T-cell engineering, positioning it as a promising transient approach for future studies.

The electroporation of activated T cells with mRNA encoding ^mfh^AXL CAR achieved a high transfection efficiency, with over 70% of the T cells expressing the ^mfh^AXL CAR for 3 days. Our study revealed that these ^mfh^AXL CAR-T cells demonstrated potent cytotoxicity against lung cancer cells within 3 days after electroporation. Furthermore, when compared to non-transfected T cells, the ^mfh^AXL CAR-T cells displayed superior, dose-dependent cytotoxicity against pancreatic cancer cells (Panc-1) and non-small cell lung cancer cells (A549). The cytotoxicity of the ^mfh^AXL CAR-T cells significantly surpassed that of non-transfected T cells. To further investigate whether the ^mfh^AXL CAR-T cells had been functionally reprogrammed to bind their specific antigen, we co-incubated these cells with cancer cells and assessed the T-cell activation. We utilized the A549 and Panc-1 cancer cells, both of which are characterized by high AXL expression, as target cells for the ^mfh^AXL CAR-T cells. Upon co-incubation with AXL-positive cancer cells, the ^mfh^AXL CAR-T cells were specifically activated to secrete IL-2 and IFN-γ, indicating their selective responsiveness to the antigen. Furthermore, we evaluated the in vivo antitumor efficacy of the ^mfh^AXL CAR-T cells. Multiple intravenous administrations of these engineered cells led to the effective control of tumor growth. Notably, the treatment was well-tolerated, as evidenced by the absence of any toxicity signs in the body weight measurements [41].

The limitation of mRNA-engineered CAR-T cells stems from their transient nature; yet, this transient characteristic can be advantageous in mitigating toxicities, such as insertional mutagenesis, on-target off-tumor toxicity, and CRS, albeit necessitating repeated administrations. In our study, mRNA encoding ^mfh^AXL CAR was electroporated into activated T cells to rapidly generate ^mfh^AXL CAR-T cells. And the short-term expression of ^mfh^AXL CAR (4–6 days) appears sufficient to elicit significant cytotoxic effects on tumor cells in vitro and to delay tumor growth in vivo through multiple infusions of ^mfh^AXL CAR-T cells. However, the potential immunogenicity and toxicity of ^mfh^AXL CAR-T cells necessitate thorough investigations in humanized immunocompetent mouse models expressing the human AXL antigen, which is challenging to assess in the current immunodeficient mouse models [42,43]. Moreover, their long-term efficacy in controlling tumor growth in clinical applications may be inadequate. Therefore, future investigations should concentrate on optimizing the sustained efficacy of ^mfh^AXL CAR-T cells. Clinical studies are necessary to demonstrate the safety and efficacy of ^mfh^AXL CAR-T cells in cancer patients, and our current study provides a compelling rationale for such investigations. Moreover, additional research is needed to explore the combination of mRNA-engineered CAR-T cells with other immunotherapeutic strategies, including cytokines and immune checkpoint inhibitors (ICIs), to augment antitumor activity [44,45].

## 5. Conclusions

We have demonstrated that mRNA-engineered fully human AXL CAR-T (^mfh^AXL CAR-T) cells exhibit potent, antigen-specific antitumor activity in vitro and in vivo. This platform offers a scalable, cost-effective alternative to viral vectors, with translational potential for solid tumor immunotherapy.

## Data Availability

The original contributions presented in this study are included in the article/Supplementary Materials. Further inquiries can be directed to the corresponding authors.

## Supplementary Materials

The following supporting information can be downloaded at https://www.mdpi.com/article/10.3390/biomedicines13040844/s1: Supplementary Table S1: The nucleotide and amino acid sequence of fully human AXL CAR. Supplementary Figure S1: Gating strategy for flow cytometric analysis. (**A**) Live T cells. (**B**) The expression of ^mfh^AXL CAR on live T cells. Supplementary Figure S2: The expression of AXL on cancer cells. The flow cytometric analysis of AXL expression on A549 (lung cancer) and Panc-1 (pancreatic cancer) cells. Supplementary Figure S3: The cytotoxicity of ^mfh^AXL CAR-T cells against AXL-negative HepG2 cells. (**A**) The expression of AXL on HepG2 (liver cancer) cells. (**B**) The cytolytic activity of AXL CAR-T cells toward HepG2 cells. The bar chart represents the mean values obtained from three independent experimental measurements. ns, not significant, comparing ^mfh^AXL CAR-T to control T cells using Student’s *t*-test. Supplementary Figure S4: The luminescence imaging of tumor-bearing mice. Statistical significance was determined using one-way ANOVA: * *p* < 0.05.

## List of Abbreviations

AXL: AXL receptor tyrosine kinase; ATCC: American Type Culture Collection; CAR-T: chimeric antigen receptor T-cell; CRS: cytokine release syndrome; EP: electroporation; FBS: fetal bovine serum; HAMA: human anti-mouse antibody; IFN-γ: interferon-γ; IL-2: interleukin-2; IL-7: interleukin-7; IL-15: interleukin-15; IVT: in vitro transcription; ICIs: immune checkpoint inhibitors; LNPs: lipid nanoparticles; LV: lentivirus; MFI: mean fluorescence intensity; MDSCs: myeloid-derived suppressor cells; PBMCs: peripheral blood mononuclear cells; RTK: receptor tyrosine kinase; RT: room temperature; RV: retrovirus; scFvs: single-chain variable fragments; TAMs: tumor-associated macrophages; TME: tumor microenvironment.
