# Supplementary material for: Novel mRNA-Engineered Fully Human CAR-T Cells Targeting AXL in Solid Tumors
Source: Biomedicines. 2025 Apr 1;13(4):844. doi: 10.3390/biomedicines13040844 (PMC12024984; doi:10.3390/biomedicines13040844)
Supplement: Supplementary file 1 [file biomedicines-13-00844-s001.zip › Supplementary Table S1.pdf]

Supplementary Table S1 **The nucleotide and amino acid sequence of fully human AXL CAR**

CAR construct	CD8SP - FLAG tag - Fully human AXL-specific scFv - CD8H - **CD8TM** - 4-1BBCD - CD3ζ
Nucleotide sequence	ATGGCCCTGCCCGTGACAGCCCTGCTGCTGCCCCTCGCCCTGCTGCTGCACGCC<br>GCCCGGCCCGATTACAAGGATGATGATGACAAGCAGGTGCAGCTGCTGGAGAGC<br>GGGGGGGGGGTCGTGCAGCCAGGGAGGAGCCTGAGGCTGAGCTGCGCCGCCAGC<br>GGGTTCACATTCTCCTCCTACGGGATGCACTGGGTGAGGCAGGCCCCCGGCAAG<br>GGGCTGGAGTGGGTGGCCGTCATCAGCTACGACGGGTCGAACAAGTACTACGCC<br>GATAGCGTGAAGGGGAGGTTCACAATCAGCCGGGATAACAGCAAGAACACCCTG<br>TACCTGCAGATGAATAGCCTGAGGGCCGAGGACACCGCCGTGTACTACTGCGCC<br>AAGGACCAGGTGCTGGCCCCTGTGGGGGGCGGGCTGTTCGACTACTGGGGCCAG<br>GGCACCCTGGTGACCGTGAGCGCCGCTGGGGGGGGGGGCAGCGGCGGCGGCGGC<br>AGCGGGGGCGGGGGGTCCGACATCCAGCTGACCCAGAGCCCCTCAAGCCTGAGC<br>GCCTCCGTGGGCGACAGGGTGACAATTACATGCCGGGCCAGCCAGAGCATTTCC<br>ACATACCTGAACTGGTACCAGCAGAAGCCCGGCAAGGCCCCCGAGAGCCTGATT<br>TTCGCCGCCTCCTCACTGGCCAGCGGGGTGCCCTCCCGGTTCAGCGGCAGCGGG<br>AGCGGCACCGACTTCACCCTGACCATCTCCAGCCTGCAGCCCGAGGATTTTGCC<br>ACCTACTACTGCCAGCAGTCCTATAGTACACCTAGAACATTTGGACCAGGCACT<br>AAGGTAGAGATTAAGacgcgtACAACAACCCCAGCCCCTAGGCCTCCAACACCA<br>GCCCCAACAATCGCTTCTCAGCCACTGTCTCTCAGACCCGAGGCTTGCCGGCCT<br>GCCGCTGGCGGGGCCGTGCACACACGGGGACTCGACTTCGCTTGCGAC**ATTTAC**<br>**ATTTGGGCCCCACTCGCTGGAACATGCGGCGTGCTCCTGCTGTCTCTGGTGATC**<br>**ACACTGTACTGC**AGAGTGAAGTTCTCTAGATCTGCTGACGCCCCAGCTTACAAG<br>CAGGGGCAGAACCAGCTGTACAACGAGCTCAACCTCGGCCGGCGCGAGGAGTAC<br>GACGTGCTCGACAAGCGGCGCGGAAGAGACCCAGAGATGGGCGGAAAGCCTAGA<br>AGAAAGAACCCTCAGGAGGGACTGTACAACGAGCTCCAGAAGGACAAGATGGCT<br>GAGGCTTACTCCGAGATTGGAATGAAGGGAGAGCGGCGCAGAGGCAAGGGGCAC<br>GACGGCCTGTACCAGGGACTGTCTACCGCCACCAAGGACACATACGACGCCCTG<br>CACATGCAGGCCCTCCCACCTAGAAGAGTGAAGTTCTCTAGATCTGCTGACGCC<br>CCAGCTTACAAGCAGGGGCAGAACCAGCTGTACAACGAGCTCAACCTCGGCCGG<br>CGCGAGGAGTACGACGTGCTCGACAAGCGGCGCGGAAGAGACCCAGAGATGGGC<br>GGAAAGCCTAGAAGAAAGAACCCTCAGGAGGGACTGTACAACGAGCTCCAGAAG<br>GACAAGATGGCTGAGGCTTACTCCGAGATTGGAATGAAGGGAGAGCGGCGCAGA<br>GGCAAGGGGCACGACGGCCTGTACCAGGGACTGTCTACCGCCACCAAGGACACA<br>TACGACGCCCTGCACATGCAGGCCCTCCCACCTAGA
Amino acid sequence	MALPVTALLLPLALLLHAARPDYKDDDDKQVQLLESGGGVVQPGRSLRLSCAAS<br>GFTFSSYGMHWVRQAPGKGLEWVAVISYDGSNKYYADSVKGRFTISRDNSKNTL<br>YLQMNSLRAEDTAVYYCAKDQVLAPVGGGLFDYWGQGTLVTVSAAGGGGSGGGG<br>SGGGGSDIQLTQSPSSLSASVGDRVTITCRASQSISTYLNWYQQKPGKAPESLI<br>FAASSLASGVPSRFSGSGSGTDFTLTISSLQPEDFATYYCQQSYSTPRTFGPGT<br>KVEIKTRTTTPAPRPPTPAPTIASQPLSLRPEACRPAAGGAVHTRGLDFACD**IY**<br>**IWAPLAGTCGVLLLSLVITLYC**RVKFSRSADAPAYKQGQNQLYNELNLGRREEY<br>DVLDKRRGRDPEMGGKPRRKNPQEGLYNELQKDKMAEAYSEIGMKGERRRGKGH<br>DGLYQGLSTATKDTYDALHMQALPPRRVKFSRSADAPAYKQGQNQLYNELNLGR<br>REEYDVLDKRRGRDPEMGGKPRRKNPQEGLYNELQKDKMAEAYSEIGMKGERRR<br>GKGHDGLYQGLSTATKDTYDALHMQALPPR

Notes:

CD8SP, signal peptide of CD8; scFv, single chain variable fragment; CD8H, hinge region of CD8; CD8TM, transmembrane region of CD8; 4-1BBCD, cytoplasmic domain of 4-1BB
