# Supplementary figures and images for: Novel mRNA-Engineered Fully Human CAR-T Cells Targeting AXL in Solid Tumors
Source: Biomedicines. 2025 Apr 1;13(4):844. doi: 10.3390/biomedicines13040844 (PMC12024984; doi:10.3390/biomedicines13040844)

**A**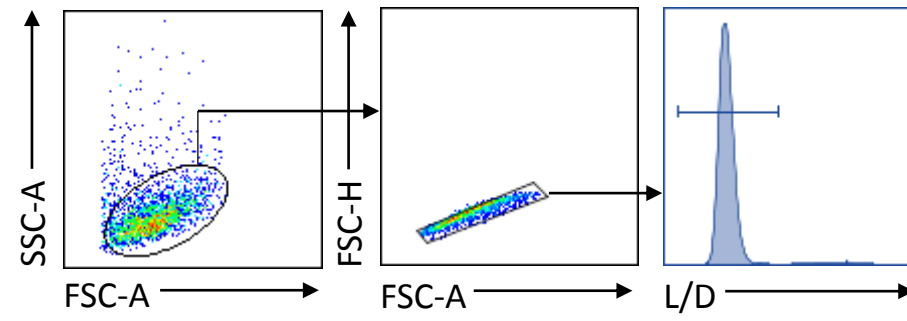**B**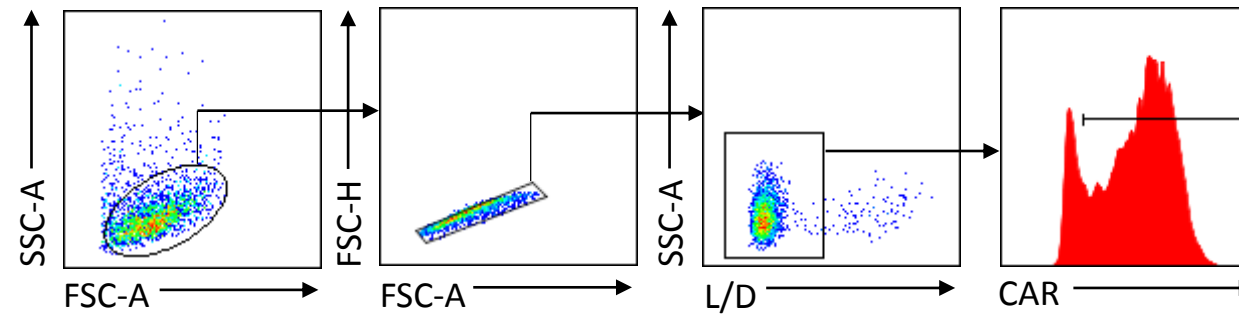

Supplement: Supplementary file 1 [file biomedicines-13-00844-s001.zip › Supplementary Figure S1.pdf]

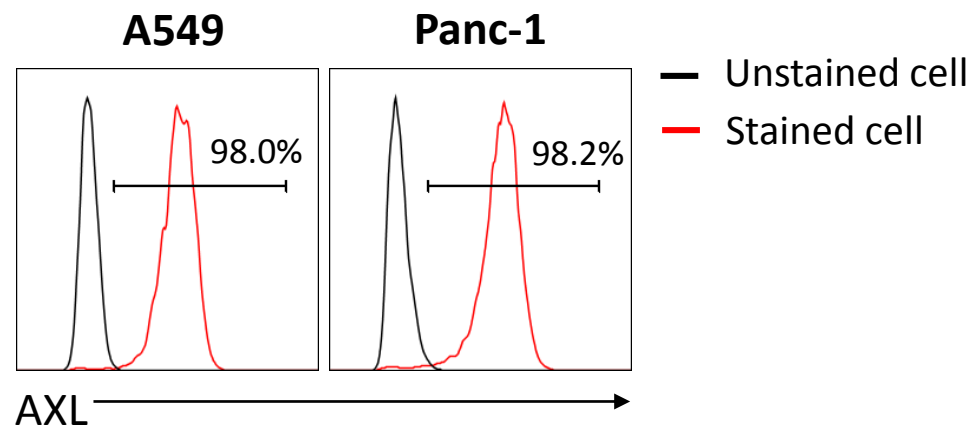

Lung cancer cell: A549

Pancreatic cancer cell: Panc-1

Supplement: Supplementary file 1 [file biomedicines-13-00844-s001.zip › Supplementary Figure S2.pdf]

**A**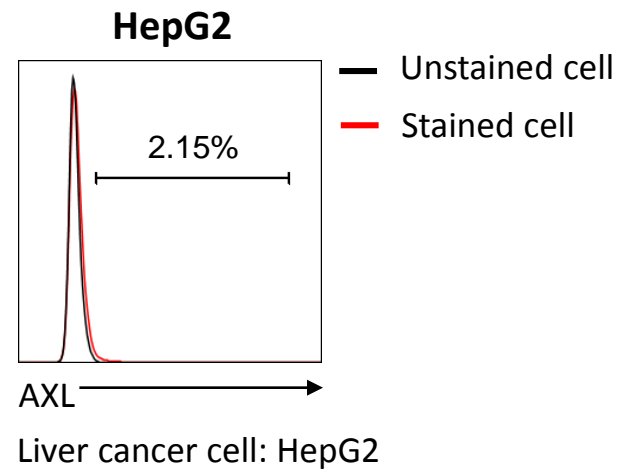**B**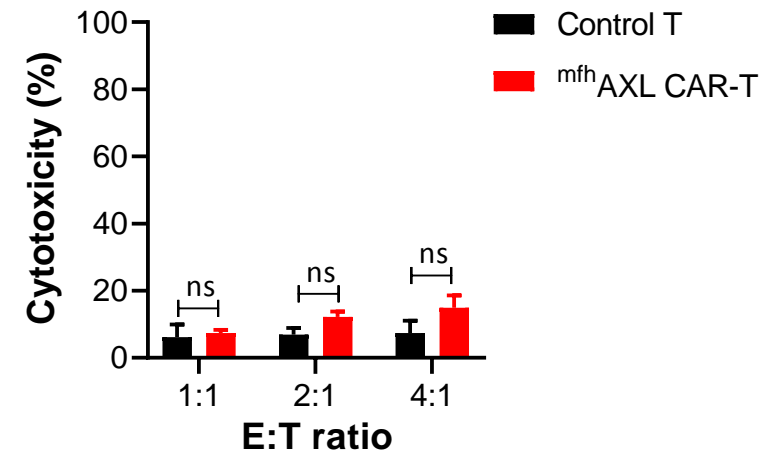

Supplement: Supplementary file 1 [file biomedicines-13-00844-s001.zip › Supplementary Figure S3.pdf]

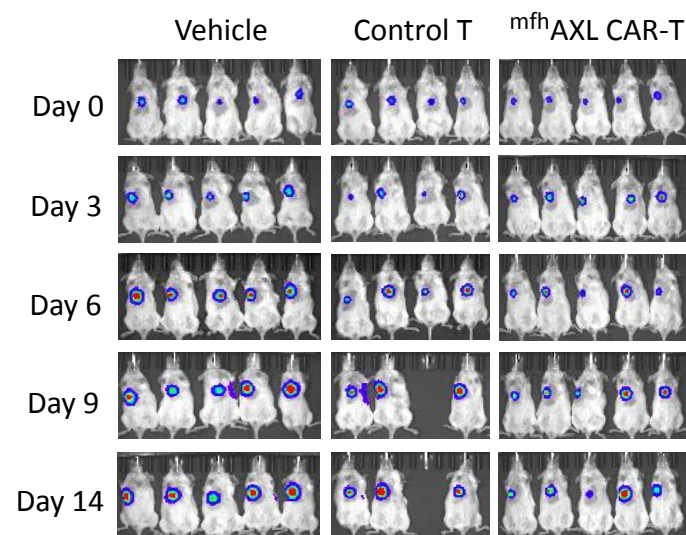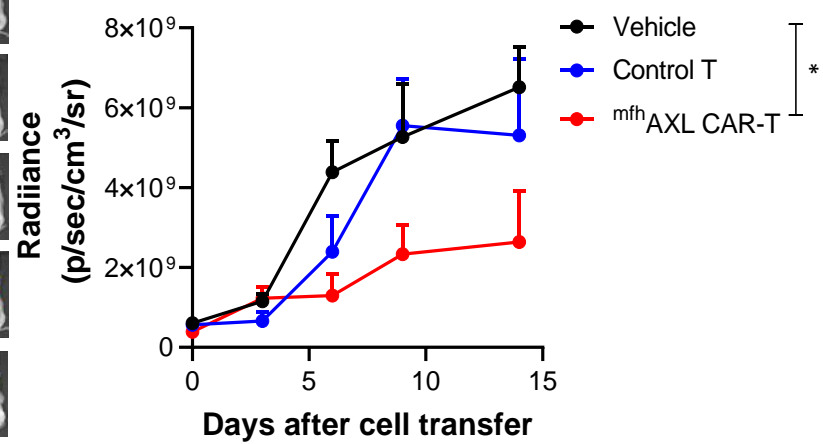

Supplement: Supplementary file 1 [file biomedicines-13-00844-s001.zip › Supplementary Figure S4.pdf]
